# Salvage mastectomy for local recurrence and second ipsilateral autologous breast reconstruction using a perforator flap from a different donor site

**DOI:** 10.1080/23320885.2018.1515020

**Published:** 2018-09-25

**Authors:** Yuki Homma, Toshihiko Satake, Kazutaka Narui, Yoshihiko Tamanoi, Mayu Muto, Takako Komiya, Shinji Kobayashi, Takashi Ishikawa, Jiro Maegawa

**Affiliations:** a Department of Plastic and Reconstructive Surgery, Yokohama City University Medical Center, Yokohama, Japan;; b Department of Breast and Thyroid Surgery, Yokohama City University Medical Center, Yokohama, Japan;; c Department of Plastic and Reconstructive Surgery, Tokyo Medical University Hospital, Tokyo, Japan;; d Department of Plastic and Reconstructive Surgery, Kanagawa Children’s Medical Center, Yokohama, Japan;; e Department of Breast Oncology and Surgery, Tokyo Medical University Hospital, Tokyo, Japan;; f Department of Plastic and Reconstructive Surgery, Yokohama City University Hospital, Yokohama, Japan

**Keywords:** Autologous breast reconstruction, DIEP flap, local recurrence, salvage mastectomy, S-GAP flap, free flap, perforator flap

## Abstract

Only one case of second ipsilateral autologous reconstruction for the same breast that had previously undergone reconstruction has been reported. Here we present a patient who underwent breast reconstruction twice using free flap from different donor sites, using a buttock after a local recurrence following the previous reconstruction with a lower abdomen.

## Introduction

Surgical resection is the primary option for local breast cancer recurrence after mastectomy and for local recurrence after breast reconstruction. Most patients who have deformed breasts after surgery for recurrence or who have disproportionate breasts express the desire to undergo a second breast reconstruction. However, presently, only one such case has been reported globally [1]. The patient had a local recurrence and underwent a second ipsilateral autologous reconstruction for the same breast that had previously undergone reconstruction after breast cancer removal. Here, we present a patient who underwent breast reconstruction using a superior gluteal artery perforator (S-GAP) flap after the identification of local recurrence following the previous reconstruction with a deep inferior epigastric perforator (DIEP) flap. We examined the applicability of a second autologous breast reconstruction.

## Case report

**Figure 1.**
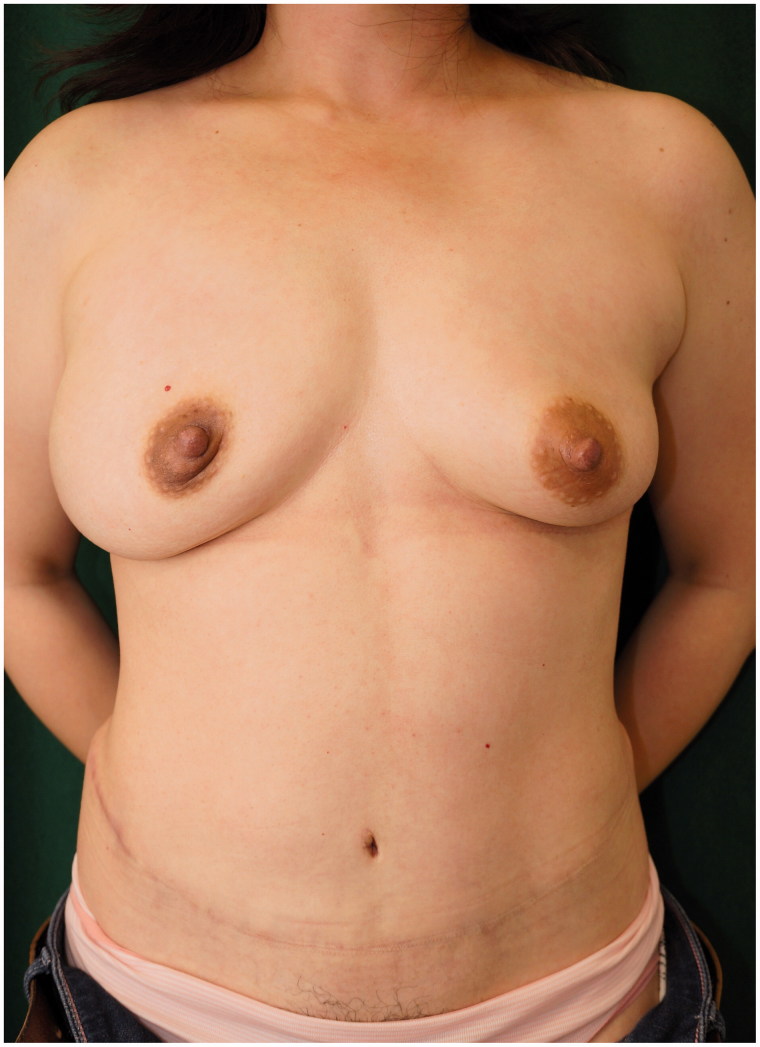
Image obtained one year after nipple-sparing mastectomy with immediate deep inferior epigastric perforator flap breast reconstruction.

**Figure 2.**
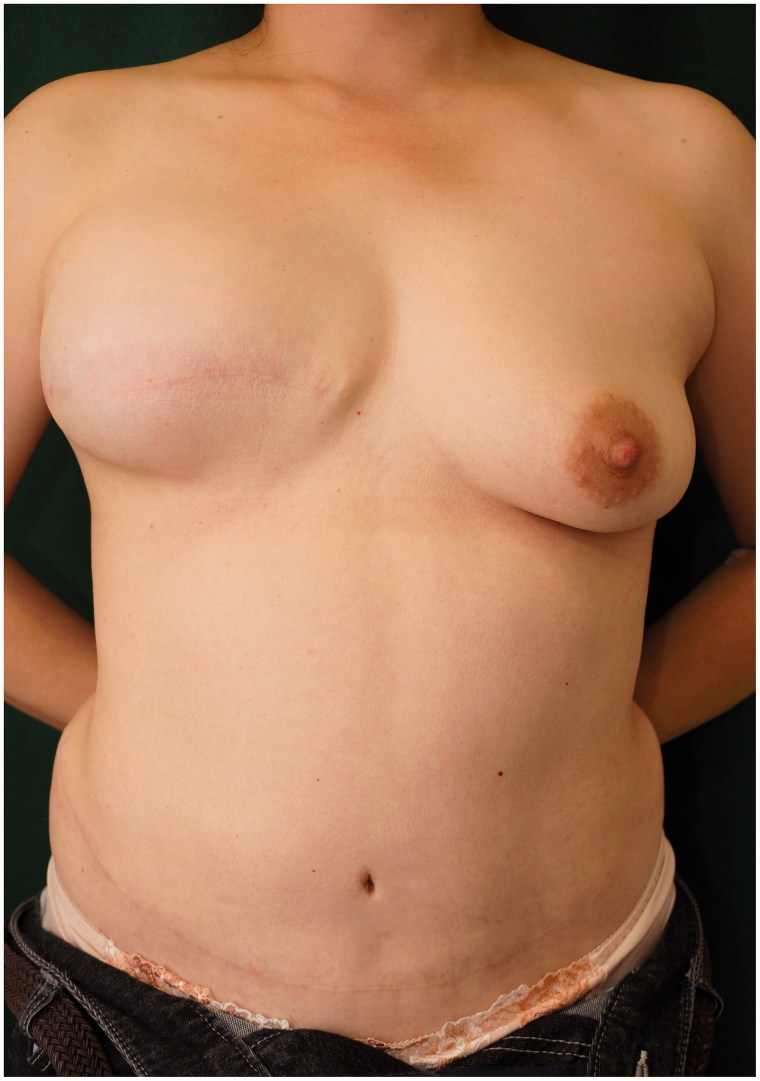
Image obtained after salvage mastectomy with simultaneous tissue expander placement.

**Figure 3.**
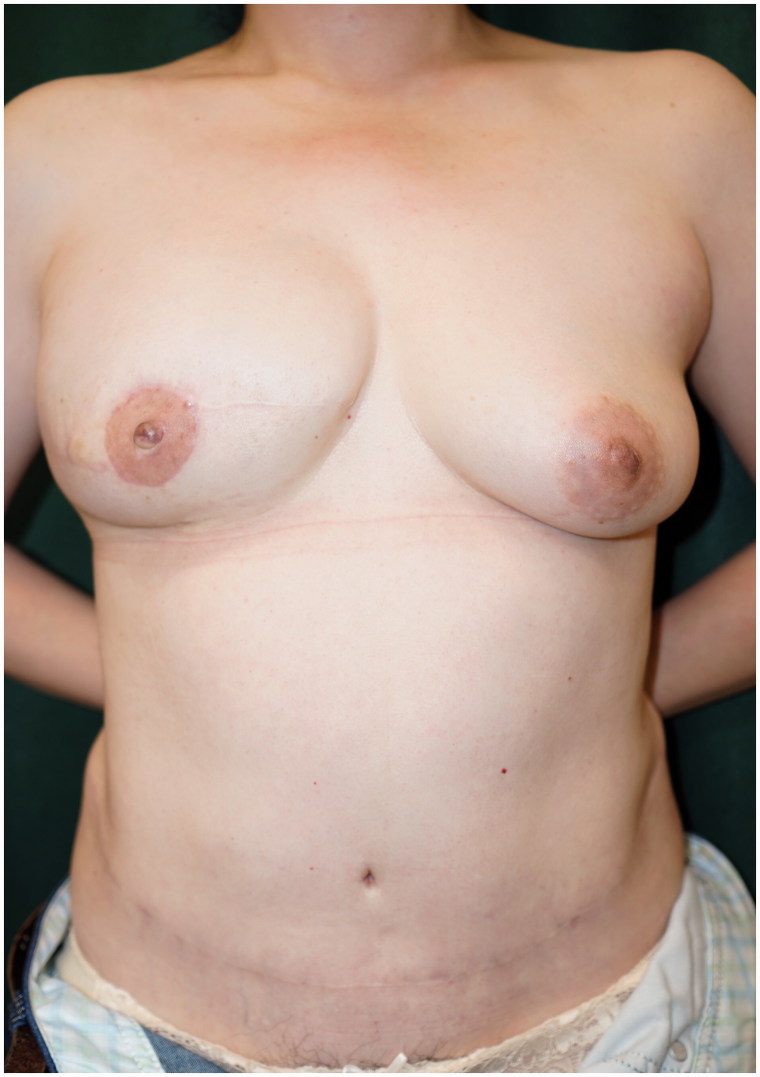
Image obtained one year after the second breast reconstruction using a superior gluteal artery perforator flap.

**Figure 4.**
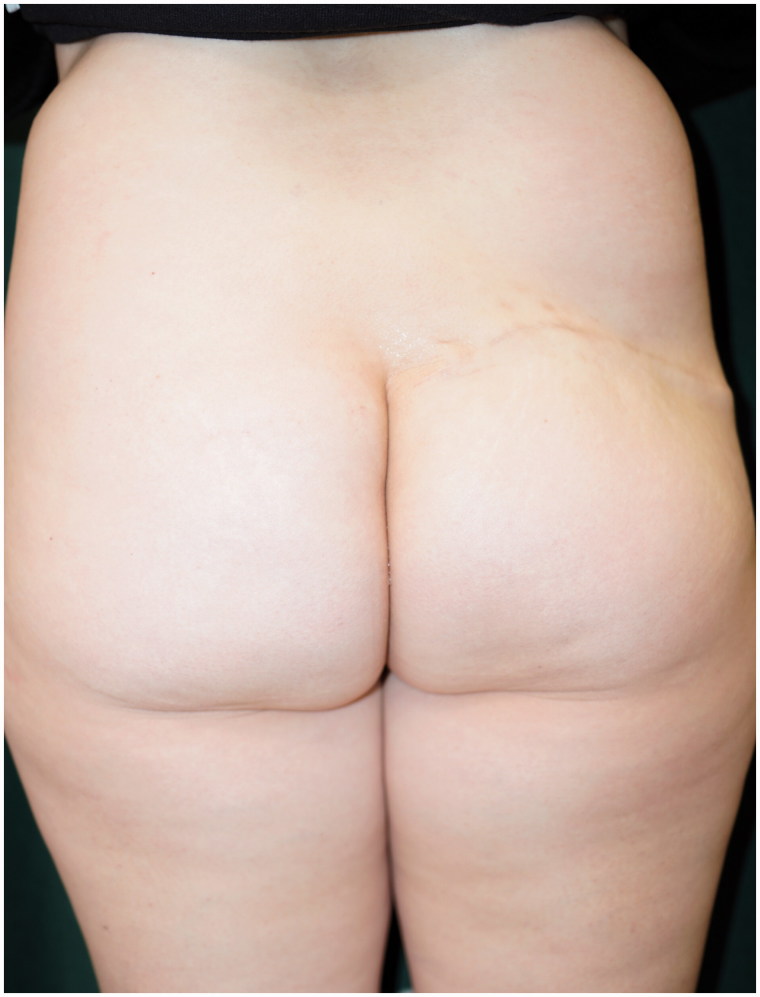
Image of the donor site one year after the second breast reconstruction.

A 43-year-old woman was diagnosed with cancer in the right breast during health screening. Magnetic resonance imaging (MRI) showed a 70 × 40 × 36-mm non-mass-like enhancement from the exterior of the right breast to the nipple region. Ductal carcinoma *in situ* (DCIS) was diagnosed on core needle biopsy; thus, mastectomy was considered necessary. As the patient desired to undergo immediate breast reconstruction, she was referred to our department. She underwent nipple-sparing mastectomy (NSM), sentinel node biopsy (SNB) performed by a breast surgeon and breast reconstruction with a de-epithelialized DIEP flap using thoracodorsal vessels as recipient vessels performed by a plastic surgeon (Figure 1). The cancer was pathologically diagnosed as DCIS. Postoperative hormone therapy was continued at a nearby medical facility. At an outpatient visit three years and eight months after surgery, a tumor measuring 5 mm was detected by palpation at the lower right region of the right breast and a tumor shadow was identified on ultrasound. Fine-needle aspiration cytology indicated the possibility of C4 local recurrence. Thus, we performed enucleation of the lesion, and a subsequent pathological examination confirmed that the tumor was invasive ductal carcinoma (IDC) with the ductal spread. In addition to the enucleated tumor, a contrast-enhanced lesion extended from the upper exterior area to just below the nipple on MRI. The patient was diagnosed with multiple cancer recurrences in the reconstructed breast. As the patient desired to undergo autologous breast reconstruction again, extensive extirpation of the nipple-areola, breast skin including the buried DIEP flap and initial breast reconstruction with an anatomical tissue expander (TE) were performed at another hospital (Figure 2). We did not give an irradiation to the breast in this case because surgical margins were free from cancer by the intensive pathological examination with 5-mm serial sectioning on the surgical specimen. At one year and eight months after insertion of the TE and postoperative adjuvant therapy with tamoxifen and leuprorelin, we extracted the TE and simultaneously performed breast reconstruction with a right S-GAP flap using internal thoracic vessels as anastomotic vessels. There has been no recurrence at two years and five months (four years after the surgery for the recurrence) postoperatively. We performed areola and nipple reconstruction through transplantation of a part of the contralateral nipple and use of a full-thickness skin graft from the proximal thigh. We have been monitoring for four years and currently, continue to monitor the patient every three months on an outpatient basis, each time giving an adjuvant therapy with leuprorelin and anastrozole. Although the right reconstructed breast appeared both upper pole fullness and lower pole skin shortage, the patient did not desire further surgery (Figures 3 and 4).

## Discussion

Free perforator flap placement and artificial approaches, such as implants and fat grafting, are some options for breast reconstruction after breast cancer removal. In free perforator flap placement, tissues from the abdomen, gluteal region or thigh are commonly used, as they provide more natural softness and mastoptosis compared with implants and provide much better volume than fat grafting. In flap placement, factors, such as age, desire to bear children, body mass index and desired breast size, are considered when selecting the donor site [2]. In our hospital, a DIEP flap is most frequently used (47.1%), followed by a GAP flap (27.9%) and a posterior medial thigh perforator (PMTP) flap [3] (25.0%) [2]. The DIEP flap is frequently used because of the adequate length of the vessel pedicle and the wide angiosome area of the free flap with the vascular pedicle originating from the deep inferior epigastric artery. In the present case, a DIEP flap was used for reconstruction in the initial surgery. However, the patient had multiple recurrences in the reconstructed breast, which required total mastectomy, and she requested for a second reconstruction. Considering that a DIEP flap had already been used in the first reconstruction, there was a need to use a flap from another body part for the second reconstruction. Alternative donor sites for autologous breast reconstruction were the gluteal and thigh regions. The thigh flap was not enough in volume for this patient. The gluteal skin color, the texture was different from the native breast skin in this patient, however, the gluteal flap seemed to provide ample amount of fibro-fatty tissue to reconstruct the breast. To avoid patchwork like appearance with gluteal skin paddle, we inserted anatomical tissue expander under the pectoralis major muscle to extend the tight breast skin envelope immediately after the salvage mastectomy. [4] In the next reconstruction, we replaced TE with a de-epithelialized S-GAP flap subcutaneously.

There is currently no established treatment guideline for breast reconstruction in cases of recurrence, and only one previous case of a second breast reconstruction for a previously reconstructed breast has been reported globally. In the previous report by Avashia et al., the second breast reconstruction was performed using a contralateral S-GAP flap [1]. However, the applicability of the reconstruction method requires further case-by-case examination. We identified recurrence cases after breast reconstruction and assessed the criteria to determine qualification for a second reconstruction (Table 1). Since 2004, we performed breast cancer surgery and breast reconstruction in four patients, and the patients did not undergo a second reconstruction for recurrence in the originally reconstructed breast. Among the four patients, two desired a second breast reconstruction. One of these patients did not qualify for reconstruction, as distant recurrences (liver and lymph node) that required whole-body treatment were noted. The other patient did not qualify for reconstruction, as she was not considered physically suitable. In the other 2 patients who did not desire reconstruction, the recurrence size was less than 15 mm and the local incision sustained aesthetics. From the findings in these patients, a second reconstruction after recurrence in a previously reconstructed breast might be considered if there is no distant recurrence, if the local recurrence area can be completely removed, if aesthetic improvement is expected, and if the patient desires reconstruction. Patterson et al. [5] and Sharma et al. [6] reported the following risk factors for cancer recurrence: 1) stage II–III, 2) tumor larger than 2 cm, 3) metastasis to the lymph nodes, 4) poorly differentiated carcinoma, 5) younger than 40 years and 6) estrogen receptor negativity. These are important factors in the decision of breast reconstruction. Aesthetic improvements after autologous breast reconstruction can increase the quality of life (QOL) of patients [7]. Nevertheless, patients should be made completely aware of not only the merits but also the demerits and risks associated with breast reconstruction. The operative time of autologous breast reconstruction is relatively long, and there is a possibility of complications, such as thrombosis, which may induce scars in areas other than the breast. In addition, the second reconstruction is relatively difficult as the best skin flap and anastomotic vessel have already been used in the first operation and the remaining options are limited. Furthermore, there are generally scars within the breast after the first surgery, which might make another surgery complex. If the breast received radiation, the tissues might be tough or blood flow might be slow, which may cause delayed healing that can adversely affect the aesthetic outcome. For such patients, a surgeon with adequate experience will be required, and this limits the selection of medical facilities.

**Table 1.** Data of patients with breast cancer recurrence at our hospital since 2004, who had not undergone a second reconstruction.

Age at the time of recurrence (years)	Pathological diagnosis and biological subtype of the original lesion	Recurrent tumor		Treatment	Reconstruction desire	Reason for not undergoing reconstruction
		No.	Maximum diameter (cm)			
43	TisNO ER(NA), HER2(NA)	1	2.5	Salvage mastectomy (reconstructed breast excision), chemotherapy	Yes	Hepatic and lymph node metastasis
39	TisNO ER(+), HER2(–)	1	7	Salvage mastectomy (reconstructed breast excision), chemotherapy	Yes	Physical status not suitable for reconstruction
56	T1N0 ER(+), HER2(–)	1	0.9	Partial excision, radiation therapy	N/A	Breast form was maintained after resection of local recurrence
28	T2N0 ER(+), HER2(–)	1	1.5			

ER: estrogen receptor; HER2: human epidermal growth factor receptor 2; N/A: not applicable.

According to the guideline, we normally perform annual screening mammography and/or ultrasonography (only ultrasonography for reconstructed breasts) after surgery, regardless of whether or not an additional reconstruction had been performed as a postoperative follow-up of breast cancer. Breast reconstruction was assumed to delay the detection of recurrence. However, the actual rate of recurrence after breast reconstruction has been reported to be 3.2–11.5% [2,5,6,8–12] (Table 2), indicating that reconstruction itself has no impact on the rate of recurrence [1,5,6,8]. The recurrence rate of the immediate autologous graft was reported as 4% and local recurrence rate of 1.5% in our hospital [13], which was relatively similar to the low recurrence rate generally recognized among other hospitals. Breast cancer recurrence after breast reconstruction is mostly in the skin and is relatively easy to detect by palpation [5]. All cases of recurrence after autologous reconstruction at our facilities have been identified through visual examination and palpation by plastic surgeons or members from the clinical oncology department during regular outpatient follow-up. Furthermore, the subject patient noticed a mass in her reconstructed breast in this case. Postoperative follow-up is important when performing breast reconstruction and we recommend patients to perform the routine self-breast examination.

**Table 2.** Recurrence rate after breast reconstruction according to previous literature.

References	Mastectomy	Reconstruction	No. of patients	Tumor stage	Follow-up (months)	Recurrence rate (%)
Simmons et al. [9]	SSM NSSM	TRAM, LD, TE, NONE	77 154	0–3	15.6 32.4	3.9 3.25
Kroll et al. [10]	SSM NSSM	TRAM	114 40	T1 or T2	72	7.0 7.5
Howard et al. [11]	?	TRAM	395	0–3	57	3.8
Gerber et al. [12]	SSM NSM MRM	LD, TRAM, implant	48 60 130	0–III	101	10.4 10.0 11.5
Linford et al. [8]	SSM after BCT	LD, TRAM, implant	60	0–III	66	10
Sharma et al. [6]	?	Autologous or implant	495	T1 or T2	89	3.2
Patterson et al. [5]	SSM SM MRM	TRAM	170 78 142	I–III	69.2	5.2 5.1 3.5
Fujimoto et al. [2]	SSM or NSM	DIEP, GAP, PMTP	136	0 − III	75	5.1

SSM: skin‐sparing mastectomy; NSSM: nipple skin‐sparing mastectomy; NSM: nipple‐sparing mastectomy; MRM: modified radical mastectomy; BCT: breast‐conserving therapy; TRAM: transverse rectus abdominis myocutaneous; LD: latissimus dorsi; TE: tissue expander; DIEP: deep inferior epigastric perforator; GAP: gluteal artery perforator; PMTP: posterior medial thigh perforator.

## Conclusions

In this case, we identified local recurrence after autologous breast reconstruction and performed a second breast cancer surgery, as well as a second ipsilateral autologous reconstruction. In the absence of a clear guideline regarding the recurrence of breast cancer, there is a need to carefully examine the treatment approaches and the applicability of reconstruction. However, as breast reconstruction itself does not have any impact on the recurrence rate and is indicated to improve a patient’s QOL, we conclude that, under certain conditions, a second reconstruction after recurrence may be considered as one of the options.
